# Differential Associations of Depressive Symptom Dimensions with Cardio-Vascular Disease in the Community: Results from the Gutenberg Health Study

**DOI:** 10.1371/journal.pone.0072014

**Published:** 2013-08-13

**Authors:** Matthias Michal, Jörg Wiltink, Yvonne Kirschner, Philipp S. Wild, Thomas Münzel, Francisco M. Ojeda, Tanja Zeller, Renate B. Schnabel, Karl Lackner, Maria Blettner, Isabella Zwiener, Manfred E. Beutel

**Affiliations:** 1 Department of Psychosomatic Medicine and Psychotherapy, University Medical Center of the Johannes Gutenberg-University Mainz, Mainz, Germany; 2 Department of Medicine II, University Medical Center of the Johannes Gutenberg-University Mainz, Mainz, Germany; 3 Department of General and Interventional Cardiology, University Medical Center Hamburg-Eppendorf, Hamburg, Germany; 4 Institute of Clinical Chemistry and Laboratory Medicine, University Medical Center of the Johannes Gutenberg-University Mainz, Mainz, Germany; 5 Institute of Medical Biostatistics, Epidemiology and Informatics, University Medical Center of the Johannes Gutenberg-University Mainz, Mainz, Germany; University of California, San Francisco, United States of America

## Abstract

A current model suggested that the somatic symptom dimension accounts for the adverse effect of depression in patients with coronary heart disease (CHD). In order to test this model we sought to determine in a large population-based sample how symptom dimensions of depression are associated with CHD, biomarkers and traditional risk factors. The associations of cognitive and somatic symptom dimensions of depression with CHD, risk factors, endothelial function, and biomarkers of inflammation and myocardial stress were analyzed cross-sectionally in a sample of n = 5000 Mid-Europeans aged 35–74 years from the Gutenberg Health Study (GHS). Only the somatic symptom dimension of depression was associated with CHD, biomarkers (inflammation, vascular function) and cardio-vascular risk factors. When multivariable adjustment was applied by demographic and cardiovascular risk factors, the weak associations of the somatic symptom dimension with the biomarkers disappeared. However, the associations of the somatic symptom dimension with CHD, myocardial infarction, obesity, dyslipidemia and family history of myocardial infarction remained. Both dimensions of depression were independently associated with a previous diagnosis of depression and distressed personality (type D). Thus, our results partly confirm current models: Somatic, but not cognitive-affective symptom dimensions are responsible for the association between depression and CHD, inflammation, vascular function and cardiovascular risk factors in the general population. However, our findings challenge the assumptions that somatic depression might be due to inflammation or vascular dysfunction as consequence of progressed atherosclerotic disease. They rather emphasize a close interplay with life-style factors and with a family history of MI.

## Introduction

Depression has been identified as a robust risk factor for the development of coronary heart disease (CHD) with an adjusted relative risk of 1.9 (95% confidence interval 1.5 to 2.4) [1], yet causal mechanisms have remained a matter of debate [2]–[4]. Potential mechanisms include an unhealthy lifestyle, poor treatment adherence, more severe underlying disease severity, autonomic and neuroendocrine imbalance, and increased inflammation [5]–[7]. To complicate matters, depressive disorders are heterogeneous, characterized by different symptom profiles, courses and disease mechanisms [4]. With respect to cardiovascular disease, several recent prospective studies showed that the somatic symptom dimension of depression (e.g. having little energy, trouble sleeping), but not the cognitive dimension (e.g. feeling down, little interest) is specifically associated with an unfavorable prognosis of CHD [4], [8]–[11]. Based on these and related findings, Ormel and de Jonge [4] have posited that post-myocardial depression is a mixture of cognitive and somatic symptom dimensions with different prototypical etiologies. According to their model, somatic factors like severity of vascular disease, atherosclerosis and systemic inflammation account for the somatic symptom dimension of depression, whereas psychological factors such as neuroticism and poor coping underly cognitive symptoms of depression. Thus, the associations between depressive symptoms and prognosis of coronary heart disease are considered mostly spurious. Irrespective of the type of depression, they argue that recurrent or persistent depression adversely affects cardiovascular prognosis by behavioral mechanisms (nonadherence with medical regimens, unhealthy lifestyle). This model was also supported by the results of a recent population-based study with n = 1261 persons aged 50–70 years, who were free of stroke and dementia [12]. The authors used the intima-media thickness of the carotid artery as a measure of atherosclerosis. They found that the association between depressive symptoms and atherosclerosis was only explained by the somatic symptom cluster of depression, but not by cognitive symptoms [12]. They concluded that depression may be an epiphenomenon of atherosclerotic disease and that symptoms originating from subclinical atherosclerotic disease inﬂate the depressive symptom score [12]. However, although several recent studies have reported that somatic symptoms of depression are better predictors of cardiac events than cognitive symptoms, this question is far from being settled as Carney and Freedland reviewed recently [13]. Notably, there is a lack of population based studies concerning this research question. In contrast to studies on samples with cardiovascular patients, in community based samples it is less likely that symptoms of depression might be inflated by underlying manifest cardiovascular disease.

Against this background the primary aims of our study are to analyze in a large sample of the general population, whether symptoms of the cognitive and somatic dimension of depression are differentially associated with cardiovascular disease and cardiovascular risk factors (e.g. dyslipidemia) on the one hand or personality and mental distress on the other hand. Our hypotheses are that somatic symptoms of depression are mainly associated with cardiovascular disease, cardiovascular risk factors, increased inflammation and vascular dysfunction, whereas cognitive symptoms are mainly associated with a medical history of depression and the distressed personality (type D) [14].

## Materials and Methods

### Study Sample

We investigated cross-sectional data of the first n = 5000 participants enrolled in the Gutenberg Health Study (GHS) from April 2007 to October 2008 [15]–[17]. The GHS is a population-based, prospective, observational single-center cohort study in the Rhein-Main-Region in western Mid-Germany. The GHS has been approved by the local ethics committee (Ethics Commission of the State Chamber of Physicians of Rhineland-Palatinate) and by the local and federal data safety commissioners. The primary aim of the study is to evaluate and improve cardiovascular risk stratification. The sample was drawn randomly from the local registry in the city of Mainz and the district of Mainz-Bingen. The sample was stratified 1∶1 for gender and residence and in equal strata for decades of age. Inclusion criteria were age 35 to 74 years and written informed consent. Persons with insufficient knowledge of German language, or physical and mental inability to participate were excluded. Based on the interim analysis 5.8% were excluded because of the exclusion criteria. The response rate (defined as the recruitment efficacy proportion, i.e. the number of persons with participation in or appointment for the baseline examination divided by the sum of number of persons with participation in or appointment for the baseline examination plus those with refusal and those who were not contactable) was 60.3%. The characteristics of the sample stratified for caseness of depression were displayed in Tables 1, 2 and 3.

**Table 1:** Sociodemographic characteristics stratified by caseness of depression.

	No Depression (ND) PHQ-9<1091.6% (n = 4580)	Depression PHQ-9≥10 7.1% (n = 357)	p
Female % (n)	48.4 (2218)	59.4 (212)	<0.0001
Age ♂, mean (age-range)*	56.1 (35 – 75)	53.3 (35 – 75)	0.0028
Age ♀, mean (age-range)*	55.1 (35 – 75)	53.0 (35 – 75)	0.0073
SES (3–21), mean (1.96 sd)	12.7 (4.0, 21.4)	11.7 (3.4, 19.9)	<0.0001
Partnership, yes, % (n)	83.2 (3810)	72.0 (257)	<0.0001

T-test for continuous variables, Chi^2^-test for categorical variables.

The sample was stratified for gender (men: women = 1∶1) and for age decades, age ranges from 35∶75 = (1∶1∶1∶1).

**Table 2:** Sample characteristics stratified by caseness of depression: Medical history and mental distress.

Dependent variables	No Depression (ND) PHQ-9<10 91.6% (n = 4580)	Depression PHQ-9≥10 7.1% (n = 357)	Logistic Regression models,OR (95% CI), p adj. for ageand sex	Linear Regression models, β-estimate (95% CI), p adj. for age and sex
**Medical History**				
CHD, % (n)	4.3 (195)	7.4 (26)	2.89 (1.83, 4.58), <0.0001	
MI, % (n)	3.0 (138)	3.7 (13)	1.87 (1.03, 3.42), 0.041	
Family history of MI, % (n)	17.3 (792)	23.0 (82)	1.42 (1.09, 1.84), 0.0083	
Diabetes, % (n)	7.2 (329)	10.1 (36)	1.95 (1.34, 2.85), 0.00052	
Hypertension, % (n)	51.3 (2351)	48.3 (172)	1.12 (0.89, 1.41), 0.35	
Treatment of hypertension, % (n)	28.4 (1300)	29.7 (106)	1.40 (1.08, 1.81), 0.011	
Dyslipidemia, % (n)	28.7 (1316)	35.0 (125)	1.55 (1.22, 1.95), 0.00024	
Smoking (current), % (n)	18.6 (849)	28.6 (102)	1.67 (1.30, 2.13), 0.0005	
BMI, mean±1.96 sd	27.1 (17.9, 36.4)	27.9 (17.0, 38.9)		1.10 (0.59, 1.60), <0.0001
Activity score, median (IQR)	7017.5 (4792.5, 9255.0)	7090.0 (4970.0, 9208.75)		−429.4 (−863.9, −5.0), 0.053
**Mental distress**				
MH of Depression, % (n)	8.6 (392)	45.6 (162)	8.82 (6.96, 11.18), <0.0001	
Type D personality, % (n)	19.3 (882)	59.0 (210)	5.83 (4.65, 7.30) <0.0001	

**Table 3:** Baseline characteristics stratified by caseness of depression: Biomarkers.

Dependent variables	No Depression (ND) PHQ-9<10 91.6% (n = 4580)	Depression PHQ-9≥10 7.1% (n = 357)	Linear Regression models,β-estimate (95% CI), p adj.for age and sex	Logistic Regression models, OR (95% CI), p adj. for age and sex
**Biomarkers** *				
*CRP≥3 mg/l, % (n)	23.4 (846)	28.8 (69)		1.35 (1.00, 1.80), 0.047
*IL-18, pg/ml, median (IQR)	227.0 (180.6, 290.4)	219.9 (172.0, 290.9)	0.0081 (−0.039, 0.055), 0.73	
*IL-1-Ra, pg/ml *, median (IQR)	316 (235.5, 417.3)	336.2 (247.6, 490.7)	0.084 (0.027, 0.14), 0.0042	
*Albumin, g/l *, mean ±1.96 sd	43.2 (37.0, 49.4)	42.5 (36.2, 48.8)	−0.74 (−1.14, −0.35), 0.0002	
Fibrinogen, mg/dl, median (IQR)	345.0 (303.0, 398.0)	350.0 (298.0, 416.0)	0.019 (−0.0030, 0.042), 0.090	
NTproBNP, pg/ml, median (IQR)	61.5 (28.3, 123.7)	64.8 (29.4, 113.4)	0.0041 (−0.12, 0.13), 0.95	
**Endothelial function**				
Systolic blood pressure before measurement of Endothelialfunction; mean ±1.96 sd	133.8 (99.3, 168.9)	130.9 (98.3, 163.4)	−0.79 (−2.50, 0.92), 0.37	
Baseline brachial artery diameter,mm, mean ±1.96 sd	4.33 (2.71, 5.96)	4.16 (2.61, 5.71)	0.00044 (−0.061, 0.062), 0.99	
Flow-mediated dilatation, % *,median (IQR)	7.13(4.41, 10.67)	7.83 (5.14, 11.44)	0.37 (−0.16, 0.92), 0.17	

Adjusted regression analysis for the independent variable depression. An odds ratio adjusted for age and sex with corresponding 95%-confidence interval and p-value is given for CRP. For the other dependent variables linear regressions have been done. In this case, the regression coefficient with corresponding 95%-confidence intervals and p-values were given.

Data presented are median (25^th/^75^th^) percentile, mean ±1.96 sd, absolute and relative frequencies of subjects.

* Subjects with a self reported influenza infection, common cold or other inflammatory diseases during the last week before examination or CRP≥10 mg/dl were excluded.

### Assessment

The 5-hour baseline-examination in the study center comprised evaluation of prevalent classical cardiovascular risk factors and clinical variables, a computer-assisted personal interview, laboratory examinations from a venous blood sample, blood pressure and anthropometric measurements. In general, all examinations were performed according to standard operating procedures by certified medical technical assistants.

### Questionnaires

Depression was measured by the Patient Health Questionnaire (PHQ-9), which quantifies the frequency of being bothered by each of the 9 diagnostic criteria of Major Depression over the past 2 weeks. Responses are summed to create a score between 0 and 27 points. A PHQ-9 sum score of ≥10 was used for the definition of caseness for depression yielding a sensitivity of 81% and a specificity of 82% for any depressive disorder [18]. The somatic and cognitive dimensions of depression were defined according to prior studies [11], [19], [20]. Four PHQ-9 items related to problems with sleep, fatigability, appetite, and psychomotor agitation/retardation were classified as somatic depressive symptoms, whereas 5 items, related to lack of interest, depressed mood, negative feelings about self, concentration problems and suicidal ideation, were classified as cognitive depressive symptoms. In the current sample the Cronbach’s alpha of the cognitive dimension was 0.71, of the somatic dimension 0.62. The intercorrelation coefficient was 0.61. Type D personality was assessed with the German version of the DS14 [21], [22]. The DS14 comprises two reliable subscales with 7 items each for negative affectivity (NA) and social inhibition (SI), rated on a 5 point Likert scale (0 false to 4 true). Type D personality is defined by a cut-off score of ≥10 on both subscales. Physical activity was inquired with the “Short Questionnaire to assess health-enhancing physical activity” (SQUASH, [23], capturing commuting, leisure time, household, work and school activities. The questionnaire does not measure energy expenditure, but indicates the habitual activity level. According to [24] sleeping, lying, sitting and standing were classified as inactivity. For analysis the physical activity score was presented in quartiles (Q1 lowest, Q4 highest quartile).

### Computer-assisted Personal Interview

During the computer-assisted personal interview participants were asked whether they had ever received the definite diagnosis of any depressive disorder (medical history of lifetime diagnosis of any depressive disorder, MH of Depression) by a physician. The presence of coronary heart disease was assessed by the question: “Were you diagnosed with a stenosis of your coronary vessels?” Cardiovascular risk factors were defined as follows: Smoking was dichotomized into non-smokers (never smoker and ex-smoker) and current smokers (occasional smoker, i.e. <1 cigarette/day, and smoker, i.e. >1 cigarette/day). Obesity was defined as a body-mass index ≥30 kg/m^2^. Diabetes was defined in individuals with a definite diagnosis of diabetes by a physician or a blood glucose level of ≥ 126 mg/dl in the baseline examination after an overnight fast of at least 8 hours or a blood glucose level of ≥ 200 mg/dl after a fasting period <8 hours. Dyslipidemia was defined as a definite diagnosis of dyslipidemia by a physician or an LDL/HDL-ratio of >3.5. Hypertension was diagnosed, if antihypertensive drugs were taken, or a mean systolic blood pressure of ≥140 mmHg (diastolic blood pressure ≥90 mmHg) in the 2^nd^ and 3^rd^ standardized measurement after 8 and 11 minutes of rest. A positive family history of myocardial infarction (FH-MI) was defined as at least one myocardial infarction in a female first-degree relative of <65 years or a male first-degree relative of <60 years. The socioeconomic status (SES) was defined according to Lampert’s and Kroll’s scores of SES with a range from 3 to 27 (3 indicates the lowest SES and 27 the highest SES) [25].

### Laboratory Analysis

Serum lipid levels (total cholesterol, triglycerides, and high-density lipoprotein cholesterol), plasma levels of C-reactive protein, fibrinogen and albumin levels were measured immediately after blood withdrawal by routine methods; low-density lipoprotein cholesterol was calculated by the Friedewald formula. All other measurements were determined in plasma or serum stored immediately after blood withdrawal and centrifugation at −80°C until analysis. The measurements were done in a blinded fashion in a single batch. IL-1-Ra and IL-18 were determined by ELISA assays (IL-1-Ra: R&D Systems, USA; IL-18: MBL International; Japan). The inter-assay and intra-assay coefficients of variation were 5.68% and 3.59% for IL-1-Ra and 13% and 6.9% for IL-18. NT-proBNP was determined by the proBNP II assay on an Elecsys 2010 system (Roche Diagnostics, Germany) with intra-assay and inter-assay coefficients of variation were 1.83% and 1.53%, respectively. The inflammatory markers were selected based on literature and availability.

### Measurement of Vascular Function

Vascular function as determined by flow-mediated dilation (FMD) was measured according to a standard protocol: After an upper arm occlusion of 5 minutes, brachial artery diameter was measured in resting conditions and after induction of local reactive hyperemia. Measurements were performed by trained technicians in dark, air-conditioned rooms after at least five minutes rest and before blood draw. Two-dimensional high-resolution ultrasonic imaging of the right brachial artery was performed on a Philips HD11XE CV ultrasound machine (Philips, Best, Netherlands) using a linear array broadband probe, L12–5 (38 mm). Baseline loops and loops recorded 60 seconds after cuff release were saved digitally and subsequently artery diameters were analyzed semi-automatically three times on an off-line reading station with a commercially available software package (Medical Imaging Applications LLC, Iowa City, Iowa). Due to refusal or insufficient data quality no FMD measurement was obtained in n = 174 participants [16].

### Statistical Analysis

Variables were reported as numbers/percentage, mean (±1.96 fold standard deviation) or median (and interquartile range (25^th/^75^th^)) as appropriate. Hence we focused in this study on the differential associations of depressive symptom dimensions the somatic and cognitive subscale of the PHQ-9 were defined as our predictors. Both predictors were entered together in each regression analyses. The main dependent variables were: Medical history of CHD, myocardial infarction (MI), diabetes, hypertension, dyslipidemia, obesity, family history of MI, current smoking, physical activity, biomarkers of inflammation (CRP, IL-1-ra, IL-18, fibrinogen, albumin), myocardial stress (NT-proBNP), vascular function as measured by brachial artery diameter and FMD, medical history of depression, and Type D personality. For the logistic and linear regression analyses (depending on the distribution of the dependent variable) serially adjusted regression models were applied. In the primary model, we adjusted for age and sex. In the second model, we additionally adjusted for the traditional cardiovascular risk factors (diabetes, hypertension, dyslipidemia, obesity, family history of myocardial infarction, current smoking, physical activity and socioeconomic status). All reported p-values correspond to 2-tailed tests. As this is an explorative study no adjustments for multiple testing have been done. P-values are given for descriptive reasons only. Due to the large number of tests, p-values should be interpreted with caution and in connection with effect estimates. For the description of the sample characteristics the sample was stratified by caseness of depression. Statistical analyses were performed using SAS for Windows 9.2 TS Level 1M0 (SAS Institute Inc.) Cary, NC, USA.

## Results

The sample characteristics stratified for caseness of depression are displayed in Tables 1, 2 and 3. A total of n = 357 participants, i.e. 7.1% of the sample, scored in the range of clinically significant depression (PHQ-9≥10). As already described elsewhere depressed participants were of younger age and lower socioeconomic status and were more likely to be affected by cardiovascular disease [26].

### Age and Sex Adjusted Associations of Somatic and Cognitive Symptom Dimensions of Depression

In order to identify the differential impact of somatic and cognitive symptom dimensions of depression on cardiovascular disease regression analyses were calculated, adjusted for age and sex (Table 4). It was found that in the general population only the somatic symptom subscale was associated with a history of CHD, MI, FH-MI, hypertension, obesity and dyslipidemia. To the contrary, the cognitive subscale showed an inverse tendency with obesity and hypertension.

**Table 4:** Age and sex adjusted associations of somatic and cognitive symptom dimensions of depression.

Dependent variables	Somatic symptom subscale (0–12)		Cognitive symptom subscale (0–15)	
	Logistic Regressionmodels, OR (95% CI),p adj. for age and sex	Linear Regression models,β-estimate (95% CI),p adj. for age and sex	Logistic Regressionmodels, OR (95% CI),p adj. for age and sex	Linear Regression models, β-estimate (95% CI), p adj. for age and sex
CHD	**1.27 (1.16, 1.38), <0.0001**		1.00 (0.92, 1.10), 0.95	
History of MI	**1.22 (1.10, 1.35), <0.0001**		0.99 (0.89, 1.11), 0.90	
Family history of MI	**1.09 (1.04, 1.14), 0.0004**		1.01 (0.96, 1.06), 0.79	
Diabetes	**1.08 (1.00, 1.16), 0.039**		1.00 (0.93, 1.08), 0.93	
Hypertension	**1.06 (1.01, 1.10), 0.0083**		**0.95 (0.91, 0.99), 0.016**	
Dyslipidemia	**1.11 (1.06, 1.15), <0.0001**		0.98 (0.94, 1.02), 0.28	
Smoking (current)	1.04 (0.99, 1.09), 0.081		1.05 (1.00, 1.10), 0.057	
Obesity (BMI ≥30)	**1.16 (1.11, 1.21), <0.0001**		**0.94 (0.90, 0.98), 0.0038**	
Physical Activity score (continuous)		38.10 (−20.56, 96.75), 0.20		9.82 (−50.15, 69.79), 0.75
MH of depression	**1.21 (1.15, 1.28), <0.0001**		**1.39 (1.32, 1.47), <0.0001**	
Type D personality	**1.12 (1.08, 1.18), <0.0001**		**1.51 (1.43,1.58), <0.0001**	
**Biomarkers** *				
CRP≥3 mg/dl (yes vs. no)	**1.08 (1.03, 1.14), 0.0021**		0.98 (0.93, 1.03), 0.40	
IL-18, pg/ml		**0.011 (0.003, 0.018), 0.018**		−0.006 (−0.014, 0.001), 0.10
IL-1-ra, pg/ml		**0.021 (0.011, 0.030), 0.0001**		−0.004 (−0.0014, 0.006), 0.41
Albumin, mg/l		**−0.098 (−0.162, −0.033), 0.0031**		0.007 (−0.058, −0.073), 0.83
Fibrinogen, mg/dl		**0.007 (0.003, 0.011), 0.0002**		−0.003 (−0.007, 0.0002), 0.07
NT-proBNP, pg/ml		−0.003 (−0.024, 0.018), 0.75		−0.012 (−0.033, 0.009), 0.29

The predictors (somatic and cognitive subscale) were entered together in each regression analyses.

* For analysis of CRP, IL-18, IL-1-ra, and Albumin subjects with a self reported influenza infection, common cold or other inflammatory diseases during the last week before examination or CRP≥10 mg/dl are excluded. All biomarkers except for Albumin and CRP were log transformed.

With respect to biomarkers of inflammation only the somatic subscale was consistently associated with elevated levels of IL-1-Ra, fibrinogen, and decreased levels of albumin. For example, each increase in the somatic subscale (range 0–12) was correlated with an increase in the fibrinogen level of 0.007 mg/dl (p = 0.0002). The cognitive subscale showed only marginal associations with IL-1-ra and albumin. Both dimensions were associated with Type D personality and with a previous diagnosis of any depressive disorder.

Concerning vascular function linear regressions with FMD, respectively the basal brachial artery diameter as dependent variables and the predictors somatic and cognitive symptom scale were calculated (see Table 5). In model 1 (adjusted for age and sex), a small positive correlation with the brachial artery diameter emerged for the somatic subscale (Beta 0.013, 95%CI 0.0022–0.023, p = 0.018) and a negative one for the cognitive subscale (Beta −0.014, 95%CI −0.025–−0.0036, p = 0.0088).

**Table 5:** Association of somatic and cognitive symptom dimensions of depression with vascular function.

	Baseline brachial artery diameter				Flow Mediated Dilatation			
	Beta	95% CI		p-value	Beta	95% CI		p-value
**Model 1**								
Somatic depression (0–12)	0.013	0.0022	0.023	**0.018**	0.0037	−0.088	0.095	0.94
Cognitive depression (0–15)	−0.014	−0.025	−0.0036	**0.0088**	0.042	−0.053	0.14	0.39
**Model 2**								
Somatic depression (0–12)	−0.0015	−0.013	0.0097	0.79	0.069	−0.034	0.17	0.19
Cognitive depression (0–15)	−0.0088	−0.020	0.0026	0.13	−0.014	−0.12	0.091	0.79

Model1 adjusted for age and sex.

Model 2 adjusted for age, sex, dyslipidemia, current smoking, diabetes, hypertension, Body mass index, 5 kg/m^2^, Activity score (Q1–Q4), FH of MI and socioeconomic status (3–27).

The predictors (somatic and cognitive subscale) were entered together in each regression analyses.

Data presented are non-standardized Betas.

Model 2 R^2^ for baseline diameter 59.6%, FMD 14.6%.

### Multivariable Adjusted Associations of Somatic and Cognitive Symptom Dimensions of Depression

In the next step, multivariable adjustments were used to test the independence of the associations from traditional risk factors (see Table 6). In these fully adjusted models the somatic subscale remained associated with CHD OR = 1.25 (1.14–1.39, p<0.0001), myocardial infarction OR = 1.22 (1.09–1.37, p<0.0008), obesity OR = 1.15 (1.09–1.21, p<0.0001), and dyslipidemia OR = 1.09 (1.04–1.14, p<0.001). There was a tendency for an association of the somatic subscale with a family history of MI (OR 1.08, 1.02–1.14, p = 0.0055). Both dimensions of depression remained independently associated with a medical history of depression and Type D personality. All associations with the biomarkers disappeared in the fully adjusted model (see Table 5 vascular function, Table 6 biomarkers of inflammation and myocardial stress).

**Table 6:** Multivariable adjusted associations of somatic versus cognitive symptom dimensions of depression.

Dependent variables	Somatic symptom subscale (0–12)		Cognitive symptom subscale (0–15)	
	Logistic Regression models,OR (95% CI), p	Linear Regressionmodels, β-estimate(95% CI), p	Logistic Regressionmodels, OR (95% CI), p	Linear Regression models, β-estimate (95% CI), p
CHD	**1.25 (1.14, 1.39), <0.0001**		1.02 (0.92, 1.13), 0.68	
History of MI	**1.22 (1.09, 1.37), <0.001**		0.99 (0.88, 1.13), 0.92	
Family history of MI#	**1.08 (1.02, 1.14), 0.0055**		1.02 (0.96, 1.07), 0.51	
Diabetes	0.97 (0.89, 1.06), 0.53		1.05 (0.96, 1.15), 0.29	
Hypertension	1.01 (0.96, 1.06), 0.77		0.96 (0.91, 1.00), 0.064	
Dyslipidemia	**1.09 (1.04, 1.14), <0.001**		0.99 (0.94, 1.04), 0.62	
Smoking (current)	1.06 (1.01, 1.12), 0.019		1.00 (0.95, 1.06), 0.92	
Obesity (BMI ≥30)	**1.15 (1.09, 1.21), <0.0001**		**0.93 (0.88, 0.98), 0.0035**	
Physical Activity score (continuous)		36.05 (−38.25, 110.36), 0.34		−27.94 (−103.68, 47.80), 0.47
Medical history of depression	**1.22 (1.15, 1.30), <0.0001**		**1.39 (1.30, 1.47), <0.0001**	
Type D personality	**1.13 (1.08, 1.19), <0.0001**		**1.51 (1.43, 1.60), <0.0001**	
**Biomarkers** *				
CRP≥3 mg/dl (yes vs. no)	1.02 (0.96, 1.09), 0.44		0.98 (0.92, 1.04), 0.45	
IL-18, pg/ml		0.0061 (−0.0024, 0.015), 0.16		−0.0056 (−0.014, 0.0030), 0.20
IL-1-ra, pg/ml		0.00067 (−0.0090, 0.010), 0.89		0.0021 (−0.0077, 0.012), 0.68
Albumin, mg/l		−0.039 (−0.11, 0.032), 0.28		−0.011 (−0.082, 0.061), 0.77
Fibrinogen, mg/dl		0.0013 (−0.0027, 0.0053), 0.53		−0.0033 (−0.0074, 0.00079), 0.11
NT-proBNP, pg/ml		0.087 (−0.015, 0.032), 0.47		−0.012 (−0.036, 0.012), 0.32

Adjusted for age, sex, dyslipidemia, current smoking, diabetes, hypertension, Body mass index, 5 kg/m^2^, Activity score (Q1–Q4), FH of MI and socioeconomic status (3–27). The predictors (somatic and cognitive subscale) were entered together in each regression analyses.

# Exclusion of subjects with CHD attenuates the relationship of the somatic symptom scale with FH of MI only slightly OR 1.06 (1.00–1.12), p = 0.0361.

* For analysis of CRP, IL-18, IL-1-ra, and Albumin subjects with a self reported influenza infection, common cold or other inflammatory diseases during the last week before examination or CRP≥10 mg/dl are excluded. All biomarkers except for Albumin and CRP were log transformed.

**Figure 1:**
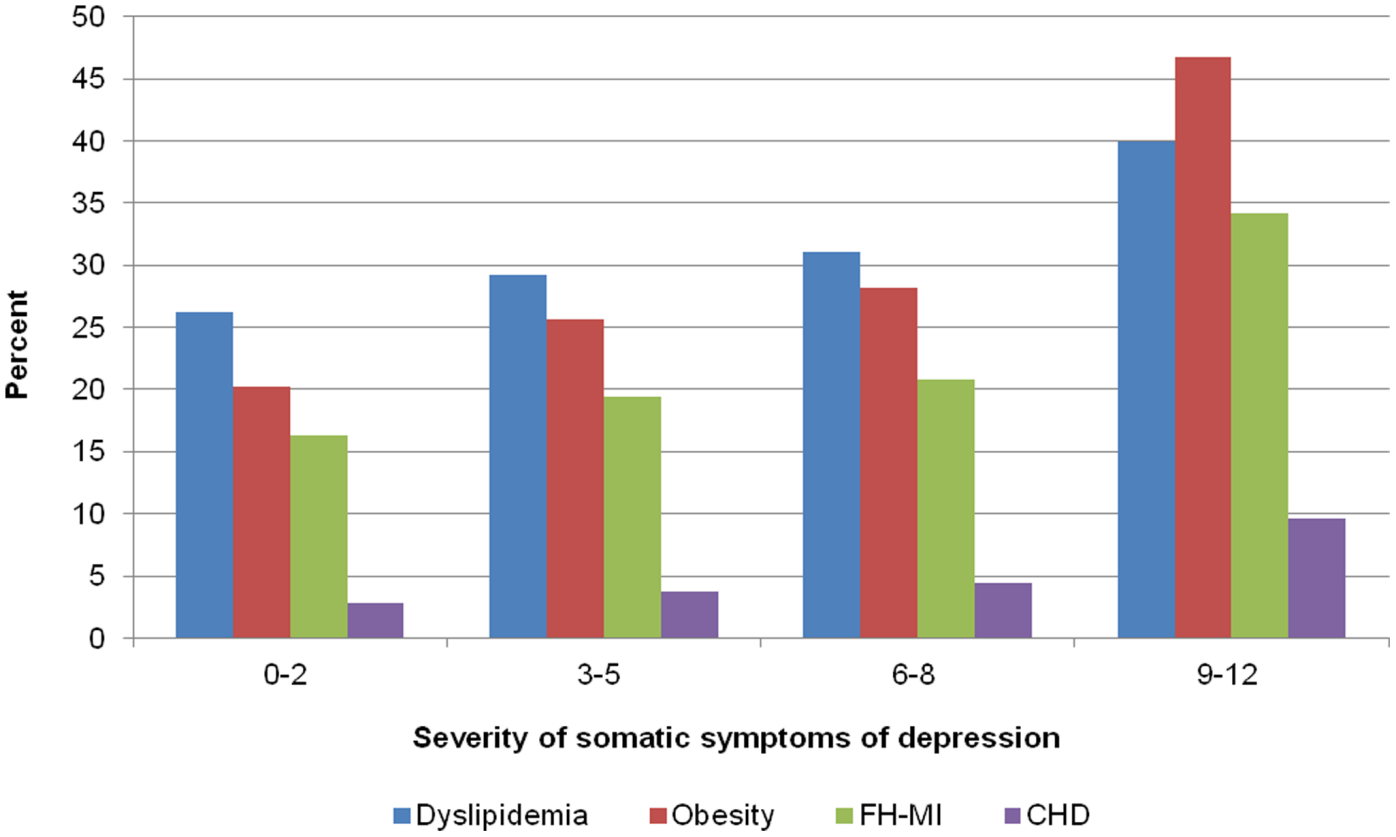
Age-standardized prevalence rates for CHD, FH-MI, obesity and dyslipidemia according to categories of severity of somatic symptoms of depression. The age standardized prevalence (according to the old European population) of the range categories of somatic symptoms of depression is 61.1%, 30.8%, 6.7% and 1.2%. In the study sample the frequency of the range categories is 62.2%, 30.5%, 6.3% and 1.1%.

Figure 1 illustrates that the frequency for CHD, FH-MI, obesity and dyslipidemia increased strongly in the sample with increasing severity of the somatic symptom dimension of depression.

## Discussion

In a large population based sample cross-sectional associations of somatic and cognitive symptom dimensions of depression with cardiovascular morbidity, biomarkers and risk factors have been analyzed. According to our hypothesis, only the somatic symptom dimension of depression accounted for these associations in the general population. Strikingly, with respect to obesity even an opposing pattern emerged. Each point on the somatic subscale accounted for an increased likelihood of being obese of 15% (OR 1.15, p<0.0001, see Table 6), whereas each point on the cognitive dimension accounted for a decreased likelihood for being obese of 7% (OR 0.93, p = 0.0035, see Table 6). This might help to explain inconsistent results on associations of obesity with mental distress [27], [28].

With respect to inflammation, only the somatic symptom dimension of depression was consistently associated with elevated levels of IL-18, IL-1-Ra, fibrinogen, CRP and decreased albumin levels. However, these associations were only of very modest effect size. For example, each point increase on the somatic subscale of depression (range 0–12) was correlated with an increase in the fibrinogen level of only 0.007 mg/dl. In consideration of standard values for fibrinogen of 180–350 mg/dl this effect lacks clinical relevance.

Concerning vascular function, we found a slight positive correlation of the somatic subscale with vascular dysfunction as measured by the baseline brachial artery diameter. According to a recent study the baseline arterial diameter correlated more strongly with classical cardiovascular risk factors than the FMD [16]. The authors concluded that the enlargement of the arterial diameter reflects structural changes of the artery, which take longer to develop and may thus mirror a sequel of long-standing exposure to cardiovascular risk factors [16]. These results, based on computations without adjustment for traditional cardiovascular risk factors, are in line with recent findings from prospective studies of cardiovascular patients, showing that the association of depression with poor cardiovascular outcome is mainly due to the somatic component of depression [4], [7], [19]. However, in consideration of a mean baseline artery diameter of 4.33 mm with an interquartile range of 2.71 to 5.95 in the group of persons without depression (see Table 3), the effect of the correlation of the somatic subscale was very small. Each point increase in the somatic subscale would be associated with a 0.013 mm increase of the baseline artery diameter only (Table 4).

When multivariable adjustment was applied for dyslipidemia, current smoking, diabetes, hypertension, Body mass index, physical activity score, family history of myocardial infarction and socioeconomic status (in addition to age and sex), the weak associations of the somatic symptom dimension with the biomarkers of inflammation and vascular dysfunction were further attenuated and lost statistical significance. Regarding biomarkers of inflammation, our findings confirm studies of patients with coronary heart disease, which could not find evidence that current depression is meaningfully associated with greater inflammation [29], [30]. Thus, our results do not support the depression-inflammation hypothesis with respect to the biomarkers assessed (CRP, IL-18, IL-1-ra, albumin, fibrinogen) [29], [30]. Concerning vascular function, our results are conflicting with several studies of patient samples with established cardiovascular disease and/or depression, which reported independent associations between depression and vascular dysfunction [31]–[37]. Contrary to these studies, our results would rather suggest that in the general population traditional risk factors (e.g. smoking, hypertension, dyslipidemia, and obesity) are more relevant for vascular function than direct effects of depression.

In contrast to biomarkers of inflammation and vascular dysfunction, somatic symptoms of depression remained robustly associated with the cardiovascular risk factors obesity and dyslipidemia. We assume bidirectional relationships between the somatic symptoms, obesity and dyslipidemia. For example, sleep disturbances as a frequent somatic symptom of depression increase the risk for obesity and dyslipidemia and vice versa [38]. Obesity and dyslipidemia are also in part intermediate endpoints of an unhealthy lifestyle, which can cause and contribute to depressive listlessness or lack of energy. Further, we found independent associations only between the somatic symptoms of depression with CHD and MI, but not for the cognitive symptom dimension. This supports the view of a specific relationship between somatic symptoms of depression with cardiovascular disease as shown in prospective patient studies [4], [7], [19]. Surprisingly, we also found an independent association of the somatic symptom dimension of depression with a family history of myocardial infarction, a finding which has not been described before up to our knowledge. Somatic symptoms of depression “predicted” that a myocardial infarction had occurred in first-degree relatives at an early age. A family history of myocardial infarction is considered as a strong genetic risk factor for developing CHD [39]. The association between somatic symptoms of depression with a positive family history of CHD may reflect both shared genetic and environmental determinants of CHD and depression [40]–[43]. In addition to the genetic influences an unhealthy sedentary life style might also be transmitted to the offspring via model learning and thus contributes to increased risk for CHD as well as for depression.

Contrary to our hypotheses not only the cognitive-affective, but also the somatic dimension of depression was consistently and strongly associated with a previous diagnosis of any depressive disorder and Type D personality. This conjoint association and the high intercorrelations of both dimensions supports the view that both symptom dimensions genuinely constitute depression, although they are differentially associated with cardiovascular disease [13].

In interpreting the results of our study several limitations have to be kept in mind. We cannot rule out, that some of the associations may have been confounded with current medication use not yet considered in our analysis. For example, it has been shown that the serotonin reuptake inhibitor Sertraline improved endothelial function and reduced inflammatory markers in CHD patients with depression [44]. Side effects of antihypertensive drugs could increase symptoms of depression [45]. The outcome medical history of CHD was based on self-report and thus may be subject to bias. As one reviewer outlined, persons with more somatic depressive symptoms could be more likely to consult a medical specialist and thus receive a diagnosis of CHD. Another limitation of our study is that we cannot preclude that the main findings may reflect differences in the severity of co-occurring somatic diseases, which have not yet been considered in our study. The unexpected lack of associations with physical activity contradicts well evidenced expectations [6] and might reflect problems of the SQUASH questionnaire, which are also reflected in the relatively high rate of missing data for this instrument. Due to the cross-sectional design of our study, causal inferences about the direction of the associations are not possible. On the other hand, we were able to include a broad range of risk factors and biomarkers in a large population-based sample.

In summary, we found that in the general population predominantly the somatic symptom dimension of depression accounted for the association of depression with CHD and related cardiovascular risk factors. It is important to note, that these associations were not only restricted to individuals with clinically significant depression but also existed at a subthreshold symptom level. The relationship between somatic symptoms of depression and CHD was maintained after adjusting for demographic and traditional risk factors. However, in contrast to recent theories, the associations between somatic depression and markers of atherosclerotic disease were only very small and did not hold up after adjusting for classic cardiovascular risk factors. Thus, life style factors such as diet, smoking and physical activity need to be considered as health risks increased by depression, and genetic and environmental effects of a family history of MI need to be disentangled.
